# Bis{μ-2,2′-[(3-aza­pentane-1,5-di­yl)bis­(nitrilo­methyl­idyne)]diphenolato}dicopper(II) dimethyl sulfoxide disolvate

**DOI:** 10.1107/S1600536808008544

**Published:** 2008-04-10

**Authors:** Guadalupe Quintero-Tellez, Carmen María González Álvarez, Sylvain Bernès, José Luis Alcántara-Flores, Yasmi Reyes-Ortega

**Affiliations:** a Centro de Química, Instituto de Ciencias, Universidad Autónoma de Puebla, Edif. 194 Complejo de Ciencias CU, San Manuel, 72570 Puebla, Pue., Mexico; b Facultad de Ciencias Químicas, Universidad Autónoma de Puebla, Edif. 179 Complejo de Ciencias CU, San Manuel, 72570 Puebla, Pue., Mexico; c DEP Facultad de Ciencias Químicas, UANL, Guerrero y Progreso S/N, Col. Treviño, 64570 Monterrey, NL, Mexico

## Abstract

The title compound, [Cu_2_(C_18_H_19_N_3_O_2_)_2_]·2C_2_H_6_OS or [Cu_2_(SalenN_3_H)_2_]·2DMSO, where SalenN_3_H is the multidentate Schiff base 2,2′-[(3-aza­pentane-1,5-di­yl)bis­(nitrilo­methyl­idyne)]diphenolate dianion and DMSO is dimethyl sulfoxide, is a solvated dinuclear Cu^II^ complex. The neutral complex is built from two Cu(SalenN_3_H) units related by an inversion center. All heteroatoms in the Schiff bases coordinate the Cu^II^ ions, which display highly distorted trigonal bipyramidal geometries. The solvent mol­ecules are located in the structural voids of the complex and are disordered over two positions with occupancies of 0.642 (15) and 0.358 (15). The previously characterized acetone disolvate of the same complex presents identical mol­ecular and crystal structures, and crystallizes with cell parameters very close to those of the DMSO disolvate reported here.

## Related literature

The title compound was synthesized by direct synthesis, using metallic copper as starting material (Gutiérrez *et al.*, 2001 ▶; Reyes-Ortega *et al.*, 2005 ▶). The same dinuclear Cu^II^ complex was previously characterized with acetone solvent in place of DMSO (McKenzie & Selvey, 1985 ▶).
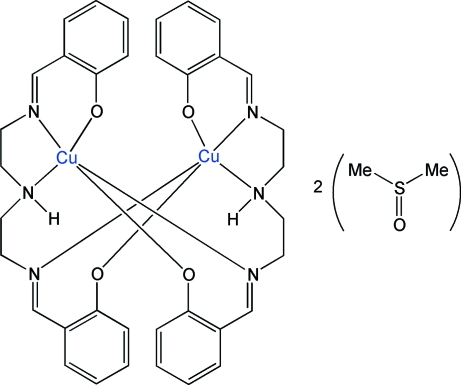


## Experimental

### 

#### Crystal data

[Cu_2_(C_18_H_19_N_3_O_2_)_2_]·2C_2_H_6_OS
*M* *_r_* = 902.06
Monoclinic, 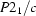

*a* = 12.817 (3) Å
*b* = 16.783 (4) Å
*c* = 9.827 (3) Å
β = 106.732 (18)°
*V* = 2024.5 (8) Å^3^
*Z* = 2
Mo *K*α radiation
μ = 1.21 mm^−1^
*T* = 298 (1) K
0.16 × 0.16 × 0.12 mm

#### Data collection

Bruker P4 diffractometer
Absorption correction: ψ scan (*XSCANS*; Siemens, 1996 ▶) *T*_min_ = 0.803, *T*_max_ = 0.866
7380 measured reflections
3599 independent reflections
2191 reflections with *I* > 2σ(*I*)
*R*_int_ = 0.076
1 standard reflections every 48 reflections intensity decay: 1%

#### Refinement

*R*[*F* ^2^ > 2σ(*F* ^2^)] = 0.062
*wR*(*F* ^2^) = 0.167
*S* = 1.05
3599 reflections
294 parameters
30 restraints
H-atom parameters constrained
Δρ_max_ = 0.47 e Å^−3^
Δρ_min_ = −0.82 e Å^−3^

### 

Data collection: *XSCANS* (Siemens, 1996 ▶); cell refinement: *XSCANS*; data reduction: *XSCANS*; program(s) used to solve structure: *SHELXTL-Plus* (Sheldrick, 2008 ▶); program(s) used to refine structure: *SHELXTL-Plus*; molecular graphics: *Mercury* (Macrae *et al.*, 2006 ▶); software used to prepare material for publication: *SHELXTL-Plus*.

## Supplementary Material

Crystal structure: contains datablocks I, global. DOI: 10.1107/S1600536808008544/si2079sup1.cif

Structure factors: contains datablocks I. DOI: 10.1107/S1600536808008544/si2079Isup2.hkl

Additional supplementary materials:  crystallographic information; 3D view; checkCIF report

## Figures and Tables

**Table:** 

Cu1—O1	1.932 (4)
Cu1—N1	1.948 (5)
Cu1—N2	2.319 (4)
Cu1—N3	1.969 (5)
Cu1—O2	1.978 (4)

**Table:** 

O1—Cu1—O2	137.2 (2)
O2—Cu1—N2	90.92 (17)
N2—Cu1—O1	131.28 (19)
N1—Cu1—N3	176.4 (2)

**Fig. 1.**
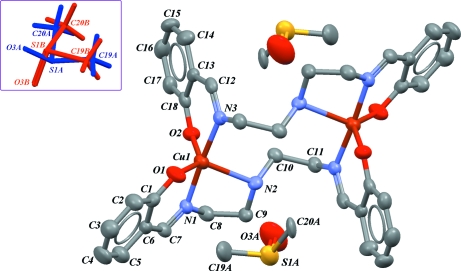
The molecular structure of the title compound. Displacement ellipsoids are given at the 40% probability level and all H atoms have been omitted. A single position for DMSO molecules has been retained, which corresponds to the main site occupancy factors, 0.642 (15). The inset represents the model refined for the disordered DMSO molecule. Site occupation factors are 0.642 (15) and 0.358 (15), for the blue and red molecule, respectively. In the main figure, non labelled atoms are generated with symmetry code 1 - x, 1 - y, 1 - z.

**Fig. 2.**
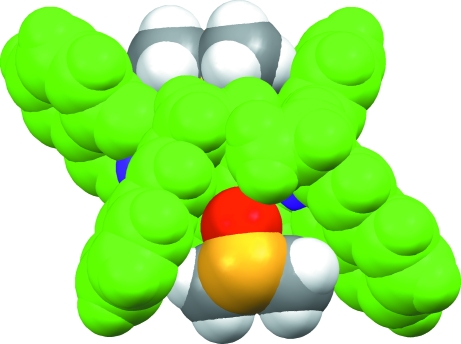
A spacefill model for the title compound. All atoms are represented, excepted less occupied disordered sites for DMSO molecules. Colours code: purple: Cu; green: Schiff bases; other: DMSO.

## supplementary crystallographic information

### Comment

The title compound was obtained during a general study about direct synthesis in coordination chemistry, *i.e*. effective synthesis of complexes using zero-valent metals, neutral ligands, and polar solvents as starting materials (Gutiérrez *et al.*, 2001). We expect that new molecular arrangements, as well as new compounds including solvent molecules as ligands, may be achieved using such reactions (Reyes-Ortega *et al.*, 2005). We are also interested in the influence of lattice solvents on magnetic properties of paramagnetic coordination compounds.

The title compound is a dinuclear centrosymmetric Cu^II^ complex, formed through coordination of two Schiff base dianions SalenN_3_H to two Cu^II^ ions; the complex is solvated by two DMSO molecules (Fig. 1). In the complex, all heteroatoms of the Schiff base ligands are coordinated to the metallic centers, which present a highly distorted trigonal-bipyramidal geometry. Atoms O1, O2 and N2 form the equatorial plane, while atoms N1 and N3 occupy apical positions, with an angle N1—Cu1—N3 = 176.4 (2)°. DMSO molecules are located in the structural voids of the complex (Fig. 2), and poorly interact with the Schiff bases, as reflected in the disorder found for this molecule (Fig. 1, inset).

The whole complex presents a rigid conformation, as ten coordination bonds are formed. It may thus be expected to be a good candidate for hosting small solvent molecules with a steric volume similar to that of DMSO. This hypothesis is, at least partially, confirmed by the previous X-ray characterization of the acetone disolvate of the same complex (McKenzie & Selvey, 1985). This compound crystallizes in the same space group, with cell parameters very close to those of the title disolvate. The complex conformation is identical, regardless of the solvent inserted in voids. For example, the non-bonding Cu···Cu separation is virtually not modified: 5.7716 (18) Å in the DMSO disolvate, *vs*. 5.809 Å in the acetone disolvate.

### Experimental

The title compound was prepared by direct synthesis, mixing equimolecular amounts (0.8 mmol) of elemental copper and neutral Schiff base SalenN_3_H_3_ in DMSO (2.4 ml). The mixture was heated at 353 K with magnetic stirring for 5.5 hrs, and then filtered. A crystalline compound, was collected after six days. Yield: 30%.

### Refinement

The asymmetric unit contains one DMSO molecule which is clearly disordered over two positions (Fig. 1, inset). S, O and C atoms were splitted over two sites. Refined occupancies converged to 0.642 (15) and 0.358 (15) for sites A and B, respectively. Geometry was regularized through restraints applied to bond lengths: S—O = 1.475 (20) and S—C = 1.750 (20) Å. Finally, sites in each pair of disordered atoms were restrained, with a standard deviation of 0.04 Å^2^, to have the same *U*_ij_ components. All H atoms were placed in idealized positions, and were allowed to ride on their carrier atoms, with C—H bond lengths fixed to 0.93 (aromatic CH), 0.97 (methylene CH_2_) or 0.96 Å (methyl CH_3_), and N—H bond length fixed to 0.91 Å. Isotropic displacement parameters for H atoms were calculated as *U*_iso_(H) = *xU*_eq_(carrier atom) where *x* = 1.5 for methyl groups and *x* = 1.2 otherwise.

### Crystal data

**Table:**

[Cu_2_(C_18_H_19_N_3_O_2_)_2_]·2C_2_H_6_OS	*D*_x_ = 1.480 Mg m^−^^3^
*M**_r_* = 902.06	Melting point: 410 K
Monoclinic, *P*2_1_/*c*	Mo *K*α radiation λ = 0.71073 Å
Hall symbol: -P 2ybc	Cell parameters from 72 reflections
*a* = 12.817 (3) Å	θ = 4.8–10.8º
*b* = 16.783 (4) Å	µ = 1.21 mm^−^^1^
*c* = 9.827 (3) Å	*T* = 298 (1) K
β = 106.732 (18)º	Cell measurement pressure: 101(2) kPa
*V* = 2024.5 (8) Å^3^	Irregular, green
*Z* = 2	0.16 × 0.16 × 0.12 mm
*F*_000_ = 940	

### Data collection

**Table:**

Bruker P4 diffractometer	2191 reflections with *I* > 2σ(*I*)
Radiation source: fine-focus sealed tube	*R*_int_ = 0.076
Monochromator: graphite	θ_max_ = 25.1º
*T* = 298(1) K	θ_min_ = 2.1º
*P* = 101(2) kPa	*h* = −14→15
2θ/ω scans	*k* = −20→20
Absorption correction: ψ scan(XSCANS; Siemens, 1996)	*l* = −11→7
*T*_min_ = 0.803, *T*_max_ = 0.866	1 standard reflections
7380 measured reflections	every 48 reflections
3599 independent reflections	intensity decay: 1%

### Refinement

**Table:**

Refinement on *F*^2^	Secondary atom site location: difference Fourier map
Least-squares matrix: full	Hydrogen site location: inferred from neighbouring sites
*R*[*F*^2^ > 2σ(*F*^2^)] = 0.062	H-atom parameters constrained
*wR*(*F*^2^) = 0.167	*w* = 1/[σ^2^(*F*_o_^2^) + (0.0556*P*)^2^ + 5.336*P*] where *P* = (*F*_o_^2^ + 2*F*_c_^2^)/3
*S* = 1.05	(Δ/σ)_max_ < 0.001
3599 reflections	Δρ_max_ = 0.47 e Å^−^^3^
294 parameters	Δρ_min_ = −0.82 e Å^−^^3^
30 restraints	Extinction correction: none
Primary atom site location: structure-invariant direct methods	

### Fractional atomic coordinates and isotropic or equivalent isotropic displacement parameters (Å^2^)

**Table:**

	*x*	*y*	*z*	*U*_iso_*/*U*_eq_	Occ. (<1)
Cu1	0.59573 (6)	0.38379 (4)	0.71636 (8)	0.0438 (3)	
O1	0.7294 (3)	0.4094 (3)	0.8606 (5)	0.0597 (12)	
O2	0.4577 (3)	0.3281 (2)	0.7063 (4)	0.0487 (11)	
N1	0.6595 (4)	0.2864 (3)	0.6677 (5)	0.0394 (11)	
N2	0.5491 (4)	0.3917 (3)	0.4705 (5)	0.0391 (11)	
H2B	0.5898	0.4311	0.4478	0.047*	
N3	0.5343 (4)	0.4857 (3)	0.7562 (5)	0.0386 (11)	
C1	0.8131 (5)	0.3647 (4)	0.9119 (6)	0.0460 (15)	
C2	0.8969 (5)	0.3907 (4)	1.0277 (7)	0.0609 (18)	
H2A	0.8908	0.4401	1.0677	0.073*	
C3	0.9878 (6)	0.3459 (5)	1.0844 (8)	0.067 (2)	
H3A	1.0419	0.3655	1.1620	0.080*	
C4	1.0014 (5)	0.2734 (5)	1.0301 (8)	0.073 (2)	
H4A	1.0636	0.2432	1.0704	0.088*	
C5	0.9219 (5)	0.2457 (4)	0.9153 (8)	0.0605 (18)	
H5A	0.9312	0.1966	0.8763	0.073*	
C6	0.8269 (5)	0.2894 (4)	0.8548 (6)	0.0427 (14)	
C7	0.7516 (5)	0.2566 (3)	0.7316 (6)	0.0433 (14)	
H7A	0.7715	0.2097	0.6951	0.052*	
C8	0.5930 (5)	0.2526 (4)	0.5346 (6)	0.0476 (15)	
H8A	0.5215	0.2383	0.5420	0.057*	
H8B	0.6272	0.2052	0.5104	0.057*	
C9	0.5838 (5)	0.3163 (3)	0.4234 (6)	0.0448 (15)	
H9A	0.6538	0.3233	0.4056	0.054*	
H9B	0.5315	0.2998	0.3353	0.054*	
C10	0.4349 (4)	0.4115 (3)	0.4060 (6)	0.0408 (14)	
H10A	0.4141	0.4527	0.4624	0.049*	
H10B	0.3910	0.3647	0.4090	0.049*	
C11	0.4088 (5)	0.4401 (3)	0.2531 (6)	0.0423 (14)	
H11A	0.4302	0.3996	0.1959	0.051*	
H11B	0.3309	0.4483	0.2153	0.051*	
C12	0.4532 (5)	0.4911 (4)	0.8083 (6)	0.0432 (15)	
H12A	0.4395	0.5411	0.8401	0.052*	
C13	0.3816 (4)	0.4273 (4)	0.8226 (6)	0.0401 (14)	
C14	0.2997 (5)	0.4461 (5)	0.8867 (7)	0.0600 (19)	
H14A	0.2965	0.4973	0.9215	0.072*	
C15	0.2255 (5)	0.3910 (5)	0.8987 (8)	0.0622 (19)	
H15A	0.1722	0.4041	0.9422	0.075*	
C16	0.2297 (6)	0.3161 (5)	0.8466 (8)	0.065 (2)	
H16A	0.1796	0.2782	0.8569	0.078*	
C17	0.3054 (5)	0.2953 (4)	0.7795 (7)	0.0532 (17)	
H17A	0.3031	0.2447	0.7403	0.064*	
C18	0.3875 (5)	0.3496 (3)	0.7685 (6)	0.0415 (14)	
S1A	0.8609 (5)	0.4752 (5)	0.4851 (8)	0.068 (2)	0.642 (15)
O3A	0.7492 (8)	0.4440 (7)	0.4334 (14)	0.126 (5)	0.642 (15)
C19A	0.921 (3)	0.449 (2)	0.665 (2)	0.084 (8)	0.642 (15)
H19A	0.9266	0.3926	0.6738	0.126*	0.642 (15)
H19B	0.8767	0.4693	0.7213	0.126*	0.642 (15)
H19C	0.9924	0.4727	0.6974	0.126*	0.642 (15)
C20A	0.845 (2)	0.5781 (8)	0.505 (2)	0.054 (5)	0.642 (15)
H20A	0.8109	0.6014	0.4137	0.081*	0.642 (15)
H20B	0.9154	0.6021	0.5447	0.081*	0.642 (15)
H20C	0.8007	0.5871	0.5670	0.081*	0.642 (15)
S1B	0.8199 (9)	0.4739 (10)	0.4918 (19)	0.082 (4)	0.358 (15)
O3B	0.8755 (13)	0.4476 (9)	0.386 (2)	0.089 (7)	0.358 (15)
C19B	0.901 (5)	0.431 (4)	0.650 (6)	0.12 (2)	0.358 (15)
H19D	0.8815	0.3758	0.6532	0.182*	0.358 (15)
H19E	0.8884	0.4584	0.7299	0.182*	0.358 (15)
H19F	0.9761	0.4351	0.6538	0.182*	0.358 (15)
C20B	0.852 (5)	0.5716 (17)	0.550 (5)	0.107 (18)	0.358 (15)
H20D	0.8245	0.6078	0.4726	0.161*	0.358 (15)
H20E	0.9299	0.5772	0.5855	0.161*	0.358 (15)
H20F	0.8200	0.5835	0.6249	0.161*	0.358 (15)

### Atomic displacement parameters (Å^2^)

**Table:**

	*U*^11^	*U*^22^	*U*^33^	*U*^12^	*U*^13^	*U*^23^
Cu1	0.0420 (4)	0.0405 (4)	0.0467 (5)	0.0053 (3)	0.0092 (3)	−0.0004 (4)
O1	0.050 (3)	0.062 (3)	0.056 (3)	0.015 (2)	−0.003 (2)	−0.018 (2)
O2	0.047 (2)	0.046 (2)	0.058 (3)	0.003 (2)	0.023 (2)	−0.002 (2)
N1	0.042 (3)	0.036 (3)	0.039 (3)	0.005 (2)	0.010 (2)	0.005 (2)
N2	0.046 (3)	0.036 (3)	0.037 (3)	0.000 (2)	0.014 (2)	−0.008 (2)
N3	0.038 (3)	0.040 (3)	0.035 (3)	0.004 (2)	0.007 (2)	0.003 (2)
C1	0.045 (3)	0.063 (4)	0.031 (3)	0.005 (3)	0.012 (3)	−0.001 (3)
C2	0.056 (4)	0.071 (5)	0.048 (4)	0.013 (4)	0.004 (3)	−0.010 (4)
C3	0.058 (4)	0.084 (6)	0.054 (4)	0.004 (4)	0.007 (4)	−0.004 (4)
C4	0.048 (4)	0.094 (6)	0.066 (5)	0.029 (4)	−0.001 (4)	0.010 (5)
C5	0.050 (4)	0.062 (4)	0.066 (5)	0.012 (3)	0.012 (4)	0.006 (4)
C6	0.041 (3)	0.051 (4)	0.038 (3)	0.009 (3)	0.015 (3)	0.009 (3)
C7	0.047 (3)	0.031 (3)	0.053 (4)	0.005 (3)	0.017 (3)	0.006 (3)
C8	0.045 (3)	0.045 (4)	0.049 (4)	0.005 (3)	0.007 (3)	−0.004 (3)
C9	0.048 (3)	0.049 (4)	0.036 (3)	0.009 (3)	0.010 (3)	−0.001 (3)
C10	0.040 (3)	0.033 (3)	0.049 (4)	0.000 (2)	0.011 (3)	−0.003 (3)
C11	0.044 (3)	0.039 (3)	0.041 (4)	0.001 (3)	0.007 (3)	0.000 (3)
C12	0.053 (4)	0.042 (3)	0.027 (3)	0.009 (3)	−0.001 (3)	−0.001 (3)
C13	0.034 (3)	0.057 (4)	0.029 (3)	0.008 (3)	0.008 (2)	0.007 (3)
C14	0.051 (4)	0.084 (5)	0.045 (4)	0.008 (4)	0.014 (3)	−0.010 (4)
C15	0.050 (4)	0.079 (5)	0.069 (5)	0.010 (4)	0.035 (4)	0.007 (4)
C16	0.055 (4)	0.077 (5)	0.064 (5)	−0.006 (4)	0.020 (4)	0.021 (4)
C17	0.050 (4)	0.055 (4)	0.051 (4)	0.000 (3)	0.010 (3)	0.014 (3)
C18	0.046 (3)	0.037 (3)	0.034 (3)	0.007 (3)	0.001 (3)	0.006 (3)
S1A	0.046 (3)	0.070 (3)	0.085 (4)	−0.009 (3)	0.014 (3)	0.012 (2)
O3A	0.057 (6)	0.100 (8)	0.201 (13)	−0.036 (6)	0.005 (7)	0.026 (8)
C19A	0.098 (16)	0.063 (14)	0.105 (14)	0.017 (15)	0.050 (11)	0.046 (11)
C20A	0.056 (8)	0.052 (7)	0.044 (12)	0.003 (6)	−0.002 (8)	0.028 (6)
S1B	0.052 (9)	0.069 (5)	0.128 (7)	−0.018 (7)	0.032 (8)	0.008 (4)
O3B	0.113 (14)	0.050 (9)	0.103 (15)	0.007 (9)	0.029 (11)	−0.011 (10)
C19B	0.12 (3)	0.11 (4)	0.16 (4)	−0.05 (2)	0.07 (3)	0.04 (3)
C20B	0.11 (3)	0.15 (3)	0.06 (3)	0.00 (2)	0.02 (2)	0.04 (2)

### Geometric parameters (Å, °)

**Table:**

Cu1—O1	1.932 (4)	C10—H10B	0.9700
Cu1—N1	1.948 (5)	C11—N3^i^	1.458 (7)
Cu1—N2	2.319 (4)	C11—H11A	0.9700
Cu1—N3	1.969 (5)	C11—H11B	0.9700
Cu1—O2	1.978 (4)	C12—C13	1.444 (8)
Cu1—Cu1^i^	5.7716 (18)	C12—H12A	0.9300
O1—C1	1.286 (7)	C13—C14	1.407 (8)
O2—C18	1.277 (7)	C13—C18	1.418 (8)
N1—C7	1.270 (7)	C14—C15	1.357 (9)
N1—C8	1.455 (7)	C14—H14A	0.9300
N2—C10	1.456 (7)	C15—C16	1.363 (10)
N2—C9	1.460 (7)	C15—H15A	0.9300
N2—H2B	0.9100	C16—C17	1.367 (9)
N3—C12	1.288 (7)	C16—H16A	0.9300
N3—C11^i^	1.458 (7)	C17—C18	1.418 (8)
C1—C2	1.392 (8)	C17—H17A	0.9300
C1—C6	1.413 (8)	S1A—O3A	1.471 (10)
C2—C3	1.363 (9)	S1A—C20A	1.756 (13)
C2—H2A	0.9300	S1A—C19A	1.766 (15)
C3—C4	1.361 (10)	C19A—H19A	0.9600
C3—H3A	0.9300	C19A—H19B	0.9600
C4—C5	1.366 (9)	C19A—H19C	0.9600
C4—H4A	0.9300	C20A—H20A	0.9600
C5—C6	1.400 (8)	C20A—H20B	0.9600
C5—H5A	0.9300	C20A—H20C	0.9600
C6—C7	1.424 (8)	S1B—O3B	1.483 (16)
C7—H7A	0.9300	S1B—C20B	1.75 (2)
C8—C9	1.509 (8)	S1B—C19B	1.76 (2)
C8—H8A	0.9700	C19B—H19D	0.9600
C8—H8B	0.9700	C19B—H19E	0.9600
C9—H9A	0.9700	C19B—H19F	0.9600
C9—H9B	0.9700	C20B—H20D	0.9600
C10—C11	1.521 (8)	C20B—H20E	0.9600
C10—H10A	0.9700	C20B—H20F	0.9600
			
O1—Cu1—O2	137.2 (2)	N2—C9—H9A	109.5
O2—Cu1—N2	90.92 (17)	C8—C9—H9A	109.5
N2—Cu1—O1	131.28 (19)	N2—C9—H9B	109.5
N1—Cu1—N3	176.4 (2)	C8—C9—H9B	109.5
O1—Cu1—N1	91.16 (18)	H9A—C9—H9B	108.1
O1—Cu1—N3	88.98 (18)	N2—C10—C11	114.2 (5)
N1—Cu1—O2	91.38 (18)	N2—C10—H10A	108.7
N3—Cu1—O2	91.00 (18)	C11—C10—H10A	108.7
N1—Cu1—N2	78.03 (17)	N2—C10—H10B	108.7
N3—Cu1—N2	99.17 (17)	C11—C10—H10B	108.7
O1—Cu1—Cu1^i^	119.32 (15)	H10A—C10—H10B	107.6
N1—Cu1—Cu1^i^	120.37 (14)	N3^i^—C11—C10	111.1 (5)
N3—Cu1—Cu1^i^	56.61 (13)	N3^i^—C11—H11A	109.4
O2—Cu1—Cu1^i^	95.61 (12)	C10—C11—H11A	109.4
N2—Cu1—Cu1^i^	42.83 (11)	N3^i^—C11—H11B	109.4
C1—O1—Cu1	128.4 (4)	C10—C11—H11B	109.4
C18—O2—Cu1	125.9 (4)	H11A—C11—H11B	108.0
C7—N1—C8	120.8 (5)	N3—C12—C13	126.6 (5)
C7—N1—Cu1	127.3 (4)	N3—C12—H12A	116.7
C8—N1—Cu1	111.7 (3)	C13—C12—H12A	116.7
C10—N2—C9	114.8 (4)	C14—C13—C18	120.1 (6)
C10—N2—Cu1	113.1 (3)	C14—C13—C12	117.0 (6)
C9—N2—Cu1	105.7 (3)	C18—C13—C12	122.8 (5)
C10—N2—H2B	107.7	C15—C14—C13	121.2 (7)
C9—N2—H2B	107.7	C15—C14—H14A	119.4
Cu1—N2—H2B	107.7	C13—C14—H14A	119.4
C12—N3—C11^i^	116.1 (5)	C14—C15—C16	119.4 (6)
C12—N3—Cu1	123.7 (4)	C14—C15—H15A	120.3
C11^i^—N3—Cu1	119.6 (4)	C16—C15—H15A	120.3
O1—C1—C2	119.9 (6)	C15—C16—C17	121.8 (7)
O1—C1—C6	123.4 (5)	C15—C16—H16A	119.1
C2—C1—C6	116.7 (6)	C17—C16—H16A	119.1
C3—C2—C1	121.9 (7)	C16—C17—C18	121.2 (7)
C3—C2—H2A	119.0	C16—C17—H17A	119.4
C1—C2—H2A	119.0	C18—C17—H17A	119.4
C4—C3—C2	121.6 (7)	O2—C18—C17	119.7 (6)
C4—C3—H3A	119.2	O2—C18—C13	124.0 (6)
C2—C3—H3A	119.2	C17—C18—C13	116.2 (6)
C3—C4—C5	118.6 (6)	O3A—S1A—C20A	104.8 (10)
C3—C4—H4A	120.7	O3A—S1A—C19A	111.1 (14)
C5—C4—H4A	120.7	C20A—S1A—C19A	99.2 (14)
C4—C5—C6	121.6 (7)	O3B—S1B—C20B	113 (2)
C4—C5—H5A	119.2	O3B—S1B—C19B	103 (3)
C6—C5—H5A	119.2	C20B—S1B—C19B	94 (3)
C5—C6—C1	119.5 (6)	S1B—C19B—H19D	109.5
C5—C6—C7	116.8 (6)	S1B—C19B—H19E	109.5
C1—C6—C7	123.5 (5)	H19D—C19B—H19E	109.5
N1—C7—C6	125.0 (5)	S1B—C19B—H19F	109.5
N1—C7—H7A	117.5	H19D—C19B—H19F	109.5
C6—C7—H7A	117.5	H19E—C19B—H19F	109.5
N1—C8—C9	106.1 (5)	S1B—C20B—H20D	109.5
N1—C8—H8A	110.5	S1B—C20B—H20E	109.5
C9—C8—H8A	110.5	H20D—C20B—H20E	109.5
N1—C8—H8B	110.5	S1B—C20B—H20F	109.5
C9—C8—H8B	110.5	H20D—C20B—H20F	109.5
H8A—C8—H8B	108.7	H20E—C20B—H20F	109.5
N2—C9—C8	110.6 (5)		
			
N1—Cu1—O1—C1	−11.8 (5)	C6—C1—C2—C3	−0.4 (10)
N3—Cu1—O1—C1	171.8 (5)	C1—C2—C3—C4	0.2 (12)
O2—Cu1—O1—C1	81.5 (6)	C2—C3—C4—C5	0.6 (12)
N2—Cu1—O1—C1	−86.8 (6)	C3—C4—C5—C6	−1.2 (11)
Cu1^i^—Cu1—O1—C1	−138.1 (5)	C4—C5—C6—C1	1.1 (10)
O1—Cu1—O2—C18	63.5 (5)	C4—C5—C6—C7	177.3 (6)
N1—Cu1—O2—C18	156.7 (5)	O1—C1—C6—C5	178.3 (6)
N3—Cu1—O2—C18	−26.1 (5)	C2—C1—C6—C5	−0.3 (9)
N2—Cu1—O2—C18	−125.3 (4)	O1—C1—C6—C7	2.4 (9)
Cu1^i^—Cu1—O2—C18	−82.6 (4)	C2—C1—C6—C7	−176.2 (6)
O1—Cu1—N1—C7	6.9 (5)	C8—N1—C7—C6	174.0 (5)
O2—Cu1—N1—C7	−130.3 (5)	Cu1—N1—C7—C6	0.6 (9)
N2—Cu1—N1—C7	139.0 (5)	C5—C6—C7—N1	176.5 (6)
Cu1^i^—Cu1—N1—C7	132.3 (5)	C1—C6—C7—N1	−7.4 (9)
O1—Cu1—N1—C8	−167.0 (4)	C7—N1—C8—C9	−116.1 (6)
O2—Cu1—N1—C8	55.8 (4)	Cu1—N1—C8—C9	58.2 (5)
N2—Cu1—N1—C8	−34.9 (4)	C10—N2—C9—C8	−102.8 (6)
Cu1^i^—Cu1—N1—C8	−41.6 (4)	Cu1—N2—C9—C8	22.5 (5)
O1—Cu1—N2—C10	−146.8 (3)	N1—C8—C9—N2	−50.9 (6)
N1—Cu1—N2—C10	132.3 (4)	C9—N2—C10—C11	−75.2 (6)
N3—Cu1—N2—C10	−50.0 (4)	Cu1—N2—C10—C11	163.4 (4)
O2—Cu1—N2—C10	41.1 (4)	N2—C10—C11—N3^i^	−63.2 (6)
Cu1^i^—Cu1—N2—C10	−56.2 (3)	C11^i^—N3—C12—C13	177.1 (5)
O1—Cu1—N2—C9	86.8 (4)	Cu1—N3—C12—C13	−12.0 (8)
N1—Cu1—N2—C9	6.0 (4)	N3—C12—C13—C14	178.2 (5)
N3—Cu1—N2—C9	−176.4 (3)	N3—C12—C13—C18	−5.2 (9)
O2—Cu1—N2—C9	−85.3 (4)	C18—C13—C14—C15	0.2 (9)
Cu1^i^—Cu1—N2—C9	177.5 (4)	C12—C13—C14—C15	176.9 (6)
O1—Cu1—N3—C12	−114.8 (5)	C13—C14—C15—C16	−0.6 (10)
O2—Cu1—N3—C12	22.3 (5)	C14—C15—C16—C17	−1.4 (11)
N2—Cu1—N3—C12	113.4 (4)	C15—C16—C17—C18	3.8 (10)
Cu1^i^—Cu1—N3—C12	118.4 (5)	Cu1—O2—C18—C17	−165.4 (4)
O1—Cu1—N3—C11^i^	55.8 (4)	Cu1—O2—C18—C13	18.2 (8)
O2—Cu1—N3—C11^i^	−167.0 (4)	C16—C17—C18—O2	179.4 (6)
N2—Cu1—N3—C11^i^	−75.9 (4)	C16—C17—C18—C13	−3.9 (9)
Cu1^i^—Cu1—N3—C11^i^	−71.0 (4)	C14—C13—C18—O2	178.5 (5)
Cu1—O1—C1—C2	−172.4 (5)	C12—C13—C18—O2	2.0 (8)
Cu1—O1—C1—C6	9.1 (9)	C14—C13—C18—C17	2.0 (8)
O1—C1—C2—C3	−179.0 (6)	C12—C13—C18—C17	−174.6 (5)

Symmetry codes: (i) −*x*+1, −*y*+1, −*z*+1.
